# Cortical Deficits are Correlated with Impaired Stereopsis in Patients with Strabismus

**DOI:** 10.1007/s12264-022-00987-7

**Published:** 2022-12-08

**Authors:** Sida Xi, Yulian Zhou, Jing Yao, Xinpei Ye, Peng Zhang, Wen Wen, Chen Zhao

**Affiliations:** 1 Department of Ophthalmology and Visual Science, Eye and ENT Hospital, Shanghai Medical College, Fudan University, Shanghai, 200031 China; 2 State Key Laboratory of Medical Neurobiology and MOE Frontiers Center for Brain Science, Institutes of Brain Science, Fudan University, Shanghai, 200031 China; 3 Key Laboratory of Myopia, Fudan University, Ministry of Health, Shanghai, 200031 China; 4 Shanghai Key Laboratory of Visual Impairment and Restoration, Fudan University, Shanghai, 200031 China; 5 Department of Radiology, Eye and ENT Hospital, Shanghai Medical College, Fudan University, Shanghai, 200031 China; 6 State Key Laboratory of Brain and Cognitive Science, Institute of Biophysics, Chinese Academy of Sciences, Beijing, 100101 China

**Keywords:** Stereopsis, Binocular disparity, Functional magnetic resonance imaging, Strabismus, Intermittent exotropia

## Abstract

In this study, we explored the neural mechanism underlying impaired stereopsis and possible functional plasticity after strabismus surgery. We enrolled 18 stereo-deficient patients with intermittent exotropia before and after surgery, along with 18 healthy controls. Functional magnetic resonance imaging data were collected when participants viewed three-dimensional stimuli. Compared with controls, preoperative patients showed hypoactivation in higher-level dorsal (visual and parietal) areas and ventral visual areas. Pre- and postoperative activation did not significantly differ in patients overall; patients with improved stereopsis showed stronger postoperative activation than preoperative activation in the right V3A and left intraparietal sulcus. Worse stereopsis and fusional control were correlated with preoperative hypoactivation, suggesting that cortical deficits along the two streams might reflect impaired stereopsis in intermittent exotropia. The correlation between improved stereopsis and activation in the right V3A after surgery indicates that functional plasticity may underlie the improvement of stereopsis. Thus, additional postoperative strategies are needed to promote functional plasticity and enhance the recovery of stereopsis.

**Supplementary Information:**

The online version contains supplementary material available at 10.1007/s12264-022-00987-7.

## Introduction

Stereopsis (depth perception), is the most advanced binocular function based on simultaneous binocular vision and fusion, which enables the perception of the world in three dimensions [1]. Stereopsis requires binocular motor alignment to achieve precise horizontal separation of two retinal images, known as binocular disparity [2, 3]. Numerous studies have demonstrated that disparity-selective neurons are widely distributed throughout the visual cortex, starting from the primary visual cortex to higher-level areas like the intraparietal sulcus (IPS) and inferior temporal areas in both the dorsal and ventral visual streams [4–7]. Owing to functional magnetic resonance imaging (MRI), a noninvasive neuroimaging technique, considerable progress has been made in clarifying the neural mechanisms underlying stereoscopic processing. Higher-level areas in the ventral and dorsal streams are responsible for three-dimensional (3D) depth perception from binocular disparity. Each stream has been reported to process different aspects of stereoscopic information [8–10]: for example, the dorsal areas V3A, V7, and IPS are involved in the disparity magnitude discrimination used for visuomotor coordination [8, 9] while the ventral visual area lateral occipital cortex (LOC) is associated with pattern discrimination and object recognition [10]. Despite extensive research on the neural mechanisms that support stereoscopic depth perception in healthy individuals, stereopsis-related pathological conditions that lead to cortical deficits have rarely been discussed.

Strabismus is a misalignment of the eyes that often leads to interocular suppression, loss of binocularity, and sensory fusion. These changes eventually cause a reduction or loss of stereopsis [11]. Thus, strabismus serves as a natural disease model to provide insights into the mechanism underlying stereoscopic deficits. General neural abnormalities associated with strabismus have been reported [12], including altered spontaneous brain activity, aberrant functional connectivity [13, 14], and abnormal interhemispheric interaction [15, 16]. We recently demonstrated that abnormal local spontaneous neural activity is correlated with stereo-deficiency in exotropia [17], indicating a potential link between functional disruption in visual areas and clinical manifestations. Nevertheless, functional MRI data in these studies were collected at the resting state in the absence of visual stimuli [12–17], and only reflected the intrinsic disturbance of brain activity [18]. However, visual stimuli are required to identify functional abnormalities in stereoscopic processing. Using disparity-defined 3D stimuli, Nasr and colleagues [19, 20] demonstrated the detrimental effect of strabismic amblyopia on the response selectivity of different neuronal clusters across the extrastriate visual cortex. They did not find any significant response to absolute and relative disparity in dorsal areas V3 and V3A in patients with strabismic amblyopia. Their conclusions provide information in terms of defining the cortical sites affected by both strabismus and amblyopia. To elucidate the disparity-encoding mechanism impaired by strabismus, more targeted research excluding the potential impact of amblyopia is needed. To date, little is known regarding whether and how stereoscopic processing in strabismus is disrupted along the two visual streams. Moreover, the relationship between cortical deficits and stereoscopic performance in strabismus remains poorly understood.

In clinical practice, stereoscopic deficits may be irreversible even after successful surgical correction of the deviation in strabismus [21]. It is suspected that a poor functional prognosis in patients after surgery may result from persistent cortical deficits [22]. On the other hand, some patients with strabismus, including those with long-standing strabismus, can fully or partially restore stereoacuity after surgery [23]. Such recovery of stereopsis is presumably mediated by cortical plasticity [24]. This cortical plasticity hypothesis is supported by the finding of microstructural reorganization and altered neural activity in patients with strabismus surgery [12, 25]. However, there is little work focusing on the changes in stereoscopic processing after strabismus surgery.

Therefore, we set out to examine whether and how neural responses for depth perception differ between stereo-deficient patients and healthy controls using dynamic random-dot stereograms (RDSs) combined with functional MRI. Subsequently, we explored cortical alterations in patients after surgical realignment, particularly in those with the improvement of stereopsis.

## Materials and Methods

### Participants

This study was approved by the Ethics Review Board of the Eye and ENT Hospital, Fudan University. In accordance with the Declaration of Helsinki, written informed consent was given by all participants (or their guardians, when applicable). We included patients with intermittent exotropia (IXT), a type of intermittent strabismus, who met the following criteria: stereoacuity > 100 arcsec, coexisting vertical deviation ≤ 5 prism diopters, Snellen best-corrected visual acuity (BCVA) better than 20/25, no other ocular diseases, no history of strabismus surgery, <6.0 diopters of myopia or hyperopia, and <1.0 diopters of anisometropia. To eliminate the potential influence of amblyopia on stereopsis, patients with amblyopia were excluded. We also recruited healthy controls who met the following criteria: Snellen BCVA better than 20/25, no signs of strabismus, and stereoacuity ≤ 40 arcsec.

### Clinical Assessments and Surgical Intervention

All participants underwent complete ophthalmic examinations, which included an assessment of BCVA, refractive errors, fundus examination, angle of deviation, fusional control, and stereopsis. Angles of deviation at distance (6 m) and near (1/3 m) after 2-h monocular occlusion were measured by the prism and alternate cover test. Fusional control was assessed using the Newcastle Control Score (NCS), which incorporates subjective (home control) and objective (clinic control) criteria into a control rating scale (ranging from 0 to 9) [26]. The home control section is an estimate of the observed frequency of squint from parental observation, while the clinic control section is an evaluation of the ability to realign the two eyes after a cover test for both near (1/3 m) and distance (6 m). An NCS score of 9 means a patient with IXT manifests squint > 50% of the time at home and presents squint spontaneously both at distance and near in the clinic. Sensory status was assessed using the Titmus Stereotest (Stereo Optical Co., Chicago, USA) at near and Worth 4-dot test at near. The binocular function (BF) score was used to extend the stereoacuity scale. The BF score is derived from the log value of the stereotest and Worth 4-Dot Test results, as previously described (Table S1) [17, 27]. All examinations were performed with sufficient optical correction for each patient. All clinical assessments were repeated in patients 6 months after surgery.

All surgeries were performed by one coauthor (C.Z.) in accordance with a standard surgical protocol. Each patient underwent one of the following procedures: bilateral lateral rectus recession or unilateral lateral rectus recession-medial rectus resection. Surgical success was defined as an alignment between 10 prism diopters of exodeviation and 5 prism diopters of esodeviation, both at distance and near. Three patients were excluded from subsequent postoperative analysis because of undercorrection. Improved stereopsis was defined as an increase in BF score of more than one grade at the follow-up examination.

### MRI Acquisition

MRI data were acquired using a 3T Siemens PRISMA scanner (Siemens Healthcare, Erlangen, Germany) with a 32-channel head coil at the Eye and ENT Hospital, Fudan University. Blood oxygen level-dependent (BOLD) functional MRI data were acquired with an echo-planar imaging pulse sequence (repetition time = 2000 ms; echo time = 30 ms; 34 slices; slice thickness = 3 mm; number of volumes per run = 128; flip angle = 76°; field of view = 240 mm × 240 mm; acquisition order = interleaved). Anatomical MRI data were collected using a high-resolution T1-weighted magnetization prepared rapid gradient echo sequence (repetition time = 2200 ms; echo time = 2.45 ms; flip angle = 9°; 176 slices; slice thickness = 3 mm; 256 × 256 matrix).

### Apparatus and Visual Stimuli

Stimuli were generated with Psychophysics Toolbox extensions [28] in MatLab (MathWorks, Natick, USA) and projected onto a rear-projection screen using a liquid crystal display projector (1024 × 768 pixel resolution, 60 Hz refresh rate). When lying in the scanner, each participant watched the screen through a mirror mounted to the head coil above their head. The viewing distance was 70 cm. The screen was calibrated with a photometer (Minolta CS-100, Konica Minolta, Inc., Tokyo, Japan).

**Fig. 1:**
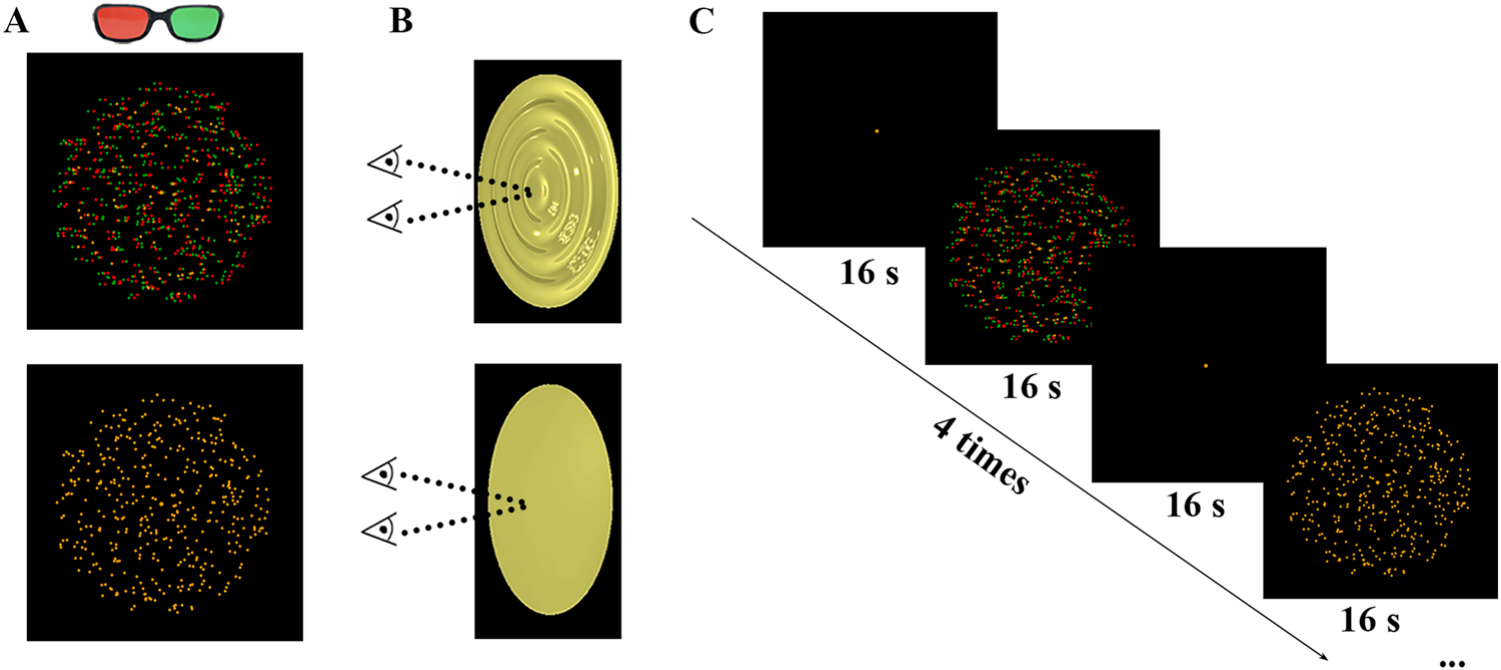
Schematic of the stimuli. **A** Stimuli are rendered as red-green spectacles for stereoscopic viewing. Upper: the “Stereo” condition is defined by disparity-varying RDSs; lower: the “Control” condition is defined by zero-disparity RDSs. **B** 3D renderings showing the perceived 3D structures (upper) or plane (lower). **C** Stimuli are presented in a block design. Each session contains 4 repetitions. In each repetition, “Stereo” and “Control” blocks are displayed in alternating order, interleaved with “Blank” blocks. 3D, three-dimensional; RDS, random-dot stereogram.

Participants wore red-green spectacles using a Kodak Wratten filter No. 25 (red) over one eye, and filter No. 58 (green) over the other to watch 16-s blocks of black background (“Blank”), disparity-varying RDS (“Stereo”), or zero-disparity RDS (“Control”). Each RDS consisted of red or green dots with 10% density (circular-like element, 15° of visual angle); the dot size was 0.12°. The luminance of the red and green dots was adjusted to 16 cd/m^2^. During “Stereo” blocks, two monocular stimuli, either red or green RDSs, were overlapped and fused; a yellow circular-wave-like pattern was thus perceived (Fig. 1A, B). The moving circular pattern varied sinusoidally in depth (±0.3°) relative to the fixation plane with a spatial frequency of 0.4 cycles/degree. The circular grating was presented at a speed of 0.5 cycles/s when viewed binocularly, similar to the approach in previous studies [29, 30]. During “Control” blocks, the positions of identical red and green RDSs were matched in both eyes; therefore, the stimulus was perceived as moving dots in the fixation plane (Fig. 1A, B). During “Blank” blocks, only a yellow central fixation spot (0.15° × 0.15°) was presented. Each run contained 4 repetitions. In each repetition, “Stereo” and “Control” blocks were displayed in alternating order; they were interleaved with “Blank” blocks (“Blank”–“Stereo”–“Blank”–“Control”) (Fig. 1C). Two runs were scanned for each participant in the whole experiment. Before scanning, participants were provided a practice run to familiarize themselves with the stimuli. Participants were instructed to remain calm and avoid unnecessary movements in the MRI scanner. During experiments, participants were asked to concentrate and maintain central fixation when viewing the stimuli, so vergence and accommodation were kept constant throughout the trial.

### MRI Data Analysis

Anatomical MRI data were analyzed using FreeSurfer 6.0 (http://surfer.nmr.mgh.harvard.edu), including cortical reconstruction and volumetric segmentation. Functional MRI data were processed using the FreeSurfer Functional Analysis Stream, version 6.0 (https://surfer.nmr.mgh.harvard.edu/fswiki/FsFast). First, the initial four functional images of each run were discarded to allow for signal stabilization. The remaining functional images were subjected to motion correction, slice timing correction, and intensity normalization. Each participant’s functional MRI data were registered to their respective anatomical volume, then resampled to an average cortical surface-based template *via* boundary-based registration [31]. Finally, spatial smoothing was applied to each surface with a Gaussian kernel (full width at half maximum = 5 mm). Surface-based cortical analyses were performed separately in each hemisphere on the average cortical surface. In the first-level analysis, a general linear model was applied to each voxel separately in each hemisphere to obtain a beta weight for every condition. Briefly, the BOLD signal for each condition (“Stereo” and “Control”) was modeled as a task regressor and convolved using a canonical hemodynamic response function (*γ* function with delay *δ* = 2.25 s and decay time constant *τ* = 1.25). The estimated head motion parameters were included in the model as nuisance regressors. Finally, beta weights for the two conditions were obtained. After comparison of beta weights among conditions, cortical activation was recorded for voxels that responded more strongly to “Stereo” blocks than to “Control” blocks.

### Group Comparison and Statistical Analysis

In the second-level group analysis, one-sample *t*-tests were used for separate examination of group mean activation in the control group and patients before and after surgery. Two-sample *t*-tests were applied to investigate group differences in cortical activation between controls and patients before and after surgery (i.e., control and preoperative; control and postoperative). Paired *t*-tests were used to assess functional changes in patients before and after surgery. A psychophysical study previously showed that surgical correction of ocular deviation does not necessarily correct visual processing deficits in the brain [32]. Therefore, we sought to investigate functional alterations in patients who had improved binocular function. As an exploratory analysis, we divided patients after surgery into two subgroups: post-improved and post-stable groups to refer to patients with improved stereopsis and stable stereopsis after surgery, respectively. Paired *t*-tests were then applied to evaluate differences between pre- and postoperative activation in the post-improved and post-stable group, respectively. Significant group-level cortical activation was corrected for multiple comparisons using a standard non-parametric permutation analysis [33]. The permutation iterations were set at a default of 10,000. It has been reported that non-parametric permutation tests can precisely control false positive rates in cluster-level inference [34]. We set the significance level to an initial uncorrected vertex-wise threshold of *P* <0.001 with family-wise error (FWE) corrected cluster *P* <0.05. A probabilistic retinotopic atlas [35] and coordinates reported in a previous study [36] were used to support the precise localization of the recorded effects.

Statistical analysis of clinical data was carried out using GraphPad Prism 8 (GraphPad Software, San Diego, USA). Demographic and clinical characteristics were compared between patient and control groups using two-sample *t*-tests, Mann–Whitney U tests, or χ^2^ tests, as appropriate. The threshold for statistical significance was set at *P* <0.05. Data are presented as the mean ± standard deviation unless otherwise stated.

### Correlation Analysis

In patients before surgery, the percentage of BOLD signal changes was extracted from clusters that exhibited significant differences between controls and patients. Relationships between cortical activation of identified clusters and clinical characteristics (e.g., age at onset, disease duration, deviation, and NCS) were assessed using Pearson correlation. Spearman correlation analysis was used to assess the relationship between cortical activation of identified clusters and BF score. In the post-improved group, the percentage of BOLD signal changes was extracted from clusters that exhibited differences between preoperative and postoperative activation. Finally, we evaluated the relationship between postoperative cortical activation of these clusters and postoperative BF score.

## Results

### Demographic and Clinical Characteristics

We recruited 18 patients (9 males; mean age, 23.4 ± 5.7 years) and 18 healthy control individuals (8 males; mean age, 23.9 ± 4.0 years). All patients enrolled were diagnosed with basic-type IXT without coexisting vertical deviation, in whom the distance deviation equaled the near deviation. As shown in Table 1, no significant difference in sex (χ^2^ test, χ^2^ = 0.33, *P* = 0.74) or age (two-sample *t*-test, *t* = 0.07, *P* = 0.95) was found between the patient and control groups. Age at onset in the patient group was 11.8 ± 5.5 years. The BF score was much worse in the patient group (3.0 ± 0.9, ~1000 arcsec) than in the control group (1.6 ± 0.1, ~40 arcsec; Mann–Whitney U test, *P* <0.0001). After surgery, a successful outcome was achieved in 15 (83.3%) of 18 patients, while 3 (16.7%) of the 18 exhibited undercorrection. Among patients with successful realignment postoperatively, 10 showed improvement (1.8 ± 0.2, ~60 arcsec) in the BF score, while 5 showed no change. The patients’ overall clinical and demographic characteristics before and after surgery are presented in Table S2.

**Table 1:** Demographic and clinical characteristics in the control group and patient group before surgery

	Patient (*n* = 18)	Control (*n* = 18)	*T*/χ^2^/M–W U Value	*P-*Value
Sex (female/male)	9/9	10/8	0.33	0.74*
Age at treatment (years)	23.4 (5.7)	22.9 (4.0)	0.07	0.95**
Age at onset (years)	11.8 (5.5)	NA	NA	NA
Duration (years)	11.7 (6.9)	NA	NA	NA
Refractive error OD (diopters)	−1.76 (2.48)	−2.46 (1.96)	0.93	0.36**
Refractive error OS (diopters)	−1.74 (2.62)	−2.40 (1.93)	0.87	0.39**
BCVA OD (logMAR)	0.01 (0.03)	−0.02 (0.05)	128	0.13†
BCVA OS (logMAR)	0.01 (0.03)	0.01 (0.04)	154	>0.99†
Exodeviation (prism diopters)	48.9 (17.7)	NA	NA	NA
Newcastle control score	5.6 (2.0)	NA	NA	NA
Binocular function score	3.0 (0.9)	1.6 (0.0)	12.50	<0.0001†

^*^P-value using Pearson’s χ^2^ test; **P-value using a two-sample *t*-test; ^†^*P-*value using a Mann–Whitney U test. Data are presented as the mean (standard deviation) unless otherwise indicated. IXT, intermittent exotropia; M–W U, Mann–Whitney U; NA, not available; BCVA, best-corrected visual acuity; logMAR, logarithm of the minimum angle of resolution; OD, right eye, OS, left eye.

### Functional Abnormalities in Patients Before Surgery

**Fig. 2:**
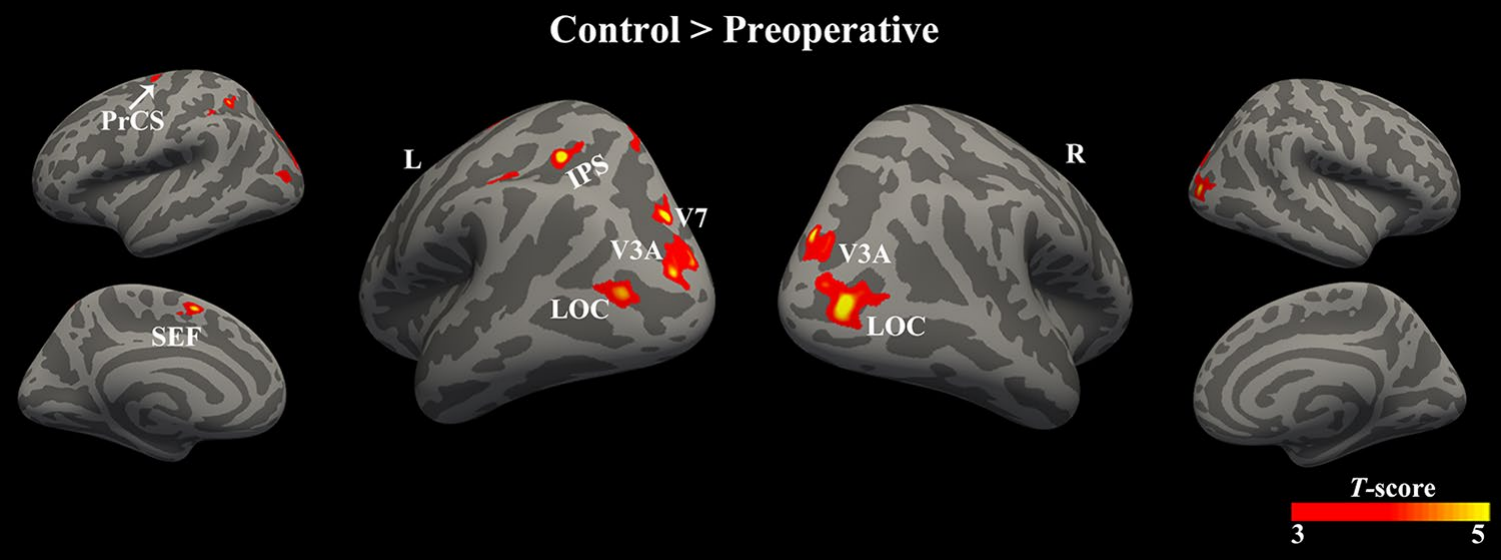
Brain areas that exhibit significant differences in cortical activation between the patient group before surgery (preoperative) and the control group. When viewing disparity-varying 3D stimuli, the patient group before surgery exhibits decreased activation in the bilateral V3A, left V7, anterior regions of the left IPS, bilateral LOC, left SEF, and left PrCS. Color scale indicates *T*-scores (permutation test, FWE corrected *P* <0.05). 3D, three-dimensional; IPS, intraparietal sulcus; LOC, lateral occipital cortex; SEF, supplementary eye field; PrCS, precentral cortex; R, right; L, left.

Group mean activation maps are shown in Fig. S1. In the control group, cortical activation evoked by 3D stimuli was widespread in the dorsal (visual and parietal) areas, higher ventral visual areas, and frontal regions. The strongest response was recorded bilaterally in V3A. In preoperative patients, the intensity and extent of activation were much smaller. Compared with healthy controls, preoperative patients exhibited decreased activation in the bilateral V3A, left V7, regions of the left IPS, bilateral LOC, left supplementary eye field (SEF), and left precentral cortex (Fig. 2, Table 2, permutation test, FWE corrected *P* <0.05).

**Table 2:** Differences in cortical activation between the control group and patient group before surgery (preoperative).

	Region	Hemi	Size (mm^2^)	Peak MNI coordinate			*T*-value	Cluster-wise *P-*value
				*x*	*y*	*z*		
*Control > Preoperative*								
Dorsal	V3A	R	427	32.2	−80.2	3.4	5.18	0.002
		L	351	−28.5	−90.5	13.2	4.50	0.004
	V7	L	143	−24	−81.2	24	5.24	0.018
	IPS	L	150	−29.9	−49.1	45	5.39	0.016
		L	120	−12.1	−66.9	59.2	3.95	0.034
		L	111	−44.7	−40.9	39.9	3.73	0.038
Ventral	LOC	R	256	19.1	−86.3	25	4.74	0.002
	LOC	L	161	−41.2	−77.4	9.1	4.28	0.014
Frontal	SEF	L	187	−9.2	4.6	57.2	5.80	0.008
	PrCS	L	117	−20.3	−16.5	60.7	4.43	0.034

The statistical threshold was set as *P* <0.001 at the voxel level with FWE corrected *P* <0.05 at the cluster level, permutation test. Hemi, hemisphere; IPS, intraparietal sulcus; LOC, lateral occipital cortex; SEF, supplementary eye field; PrCS, precentral sulcus; R, right; L, left; MNI, Montreal Neurological Institute.

### Functional Plasticity in Patients After Surgery

**Fig. 3:**
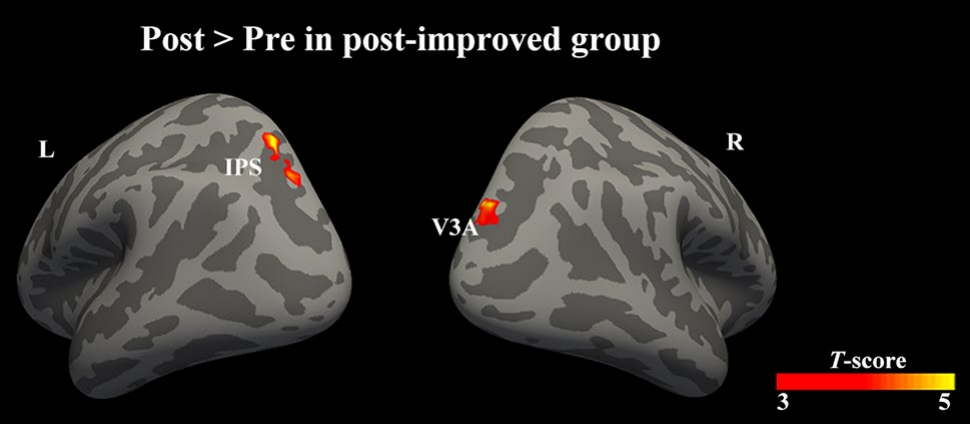
Brain areas that exhibit significant differences between postoperative and preoperative activation in the post-improved group. Patients with improved BF scores show stronger postoperative activation than preoperative activation in the right V3A and a region of the left IPS. Color scale indicates *T*-scores (permutation test, FWE corrected *P* <0.05). BF, binocular function; IPS, intraparietal sulcus; R, right; L, left.

Only 15 patients with successful surgical realignment were included in the pairwise comparison. The between-group comparison showed a statistically significant positive difference in cortical response of the right LOC between controls and patients after surgery (Fig. S2, Table S3, permutation test, FWE corrected *P* <0.05). Despite a greater extent of cortical activation in patients after surgery (Fig. S1), pairwise comparison between pre- and postoperative activation did not reveal significant differences in patients after surgery. Approximately one-third of patients after surgery showed no improvement in BF score. The absence of a significant difference is presumably because those patients had a stable sensory status after surgery [32]. For exploratory purposes, we identified a post-improved group of 10 patients with an improved BF score, and a post-stable group of 5 patients showing no change in stereopsis after surgery. Interestingly, the further pairwise comparison showed stronger postoperative activation than preoperative activation in the right V3A and left IPS in the post-improved group (Fig. 3, Table 3, permutation test, FWE corrected *P* <0.05). However, patients in the post-stable group reported no difference between pre- and postoperative activation.

**Table 3:** Differences between pre- and postoperative activation in the post-improved group^†^.

	Region	Hemi	Size (mm^2^)	Peak MNI coordinate			*T*-value	Cluster-wise *P-*value
				*x*	*y*	*z*		
Dorsal	V3A	R	226.6	23.4	−86.2	28.3	4.398	0.004
	IPS	L	104.48	−17.2	−64.6	47.4	4.895	0.002
		L	93.64	−21.6	−71.9	34.2	3.844	0.018

^†^Only stronger postoperative activation than preoperative activation was found in the post-improved group. The statistical threshold was set as *P* <0.001 at the voxel level with FWE corrected *P* <0.05 at the cluster level, permutation test. Hemi, hemisphere; IPS, intraparietal sulcus; R, right; L, left; MNI, Montreal Neurological Institute.

### Correlation Analysis

**Fig. 4:**
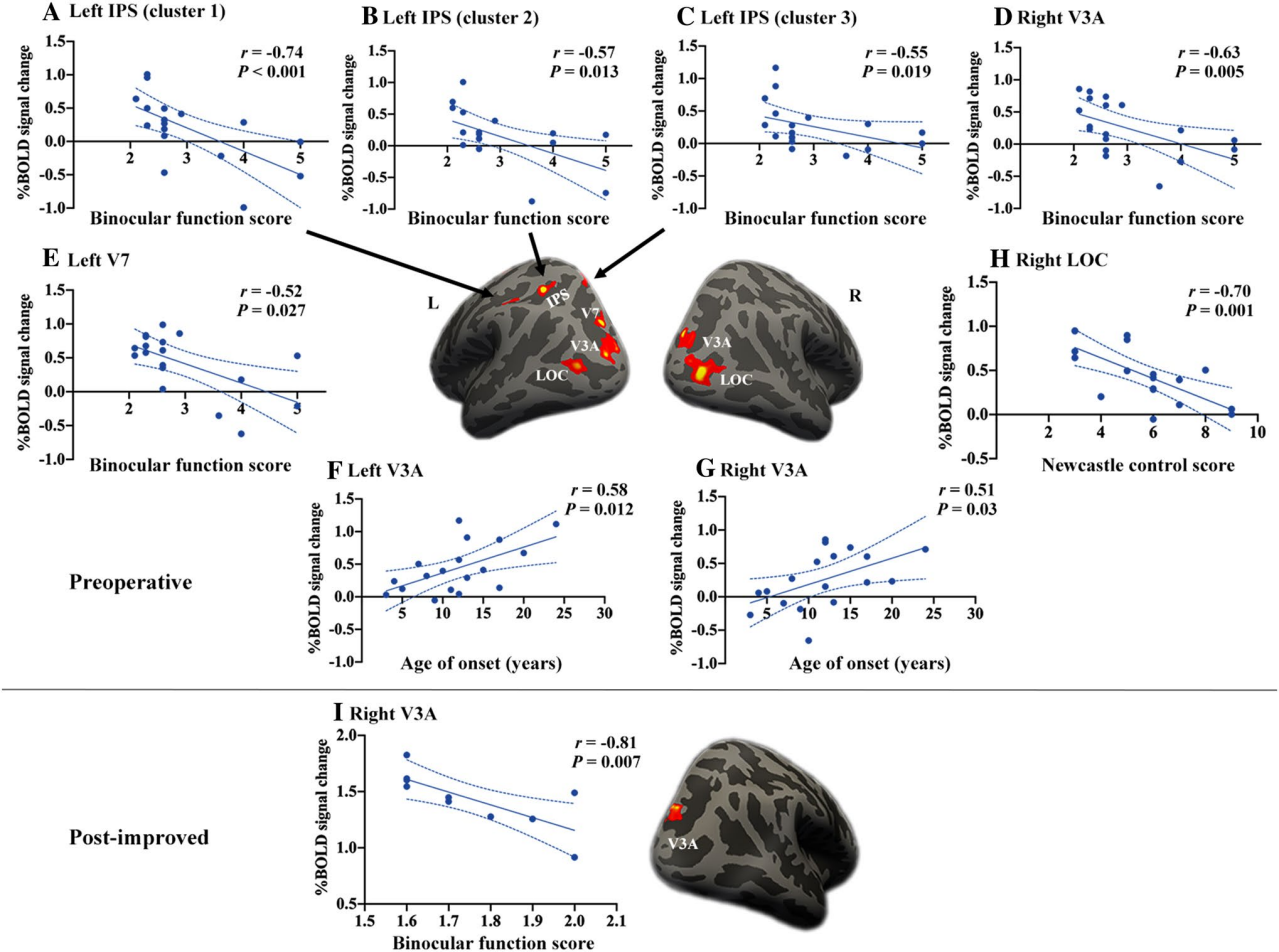
Correlations between cortical activation and clinical characteristics. In patients before surgery (preoperative, **A–E**), BF score is negatively correlated with cortical activation in the right V3A, left V7, and regions of the left IPS (clusters 1–3); **F, G** Age at strabismus onset is positively correlated with cortical activation in the bilateral V3A. **H** Newcastle Control Score is negatively correlated with cortical activation in the right LOC. **I** Postoperative BF score is negatively correlated with cortical activation in the right V3A in the post-improved group. BF, binocular function; IPS, intraparietal sulcus; LOC, lateral occipital cortex; R, right; L, left.

In patients before surgery, the BF score was strongly negatively correlated with cortical activation in the higher dorsal areas including the right V3A (*r* = −0.63, *P* = 0.005, Fig. 4D), left V7 (*r* = −0.52, *P* = 0.027, Fig. 4E), and regions of the left IPS (*r* = −0.74, *P* <0.001; *r* = −0.57, *P* = 0.013; *r* = −0.55, *P* = 0.019; Fig. 4A–C). Age at onset was positively correlated with cortical activation in the bilateral V3A in the patient group (*r* = 0.51, *P* = 0.03; *r* = 0.58, *P* = 0.012, Fig. 4F, G). Moreover, the NCS was negatively correlated with cortical activation in the right LOC in the patient group (*r* = −0.70, *P* = 0.001, Fig. 4H). After surgery, the BF score was negatively correlated with cortical activation in the right V3A in the post-improved group (*r* = −0.81, *P* = 0.007, Fig. 4I).

## Discussion

In the present study, preoperative patients showed extensive hypoactivation across occipital, parietal, and frontal cortices, compared with healthy controls. Analysis of postoperative patients did not reveal significant changes in activation after surgery (compared with preoperative values). However, stronger postoperative activation (compared with preoperative activation) was recorded in higher dorsal areas in the post-improved group. Correlation analysis revealed that the BF score and age at onset were correlated with cortical activation in higher dorsal areas and the NCS was negatively correlated with cortical activation in the right LOC in patients before surgery. Furthermore, the post-improved group exhibited a significant correlation between the postoperative BF score and cortical activation in the right V3A.

Experimental models of strabismus in non-human primates have verified disparity-selective neurons in V3A and highlighted the importance of V3A in disparity computations [37, 38]. Human neuroimaging studies have clarified the roles of V3A and V7 in stereoscopic processing [9, 39]. In addition, Ng *et al*. [40] demonstrated disparity-specific feedforward connectivity between V3A and V7. Consistent with the reduced activation that our patient group exhibited in the bilateral V3A and left V7, previous studies of strabismus have revealed the absence of functional MRI responses to different disparity cues [19] and loss of binocular interaction in V3A in affected patients [41]. In addition, patients before surgery exhibited reduced activation in anterior regions of the left IPS, which are known to extract and process 3D shapes from both binocular and monocular depth cues [8, 42]. The IPS is responsible for controlling eye movement and eye-hand coordination [43]. Reduced cortical thickness and decreased white matter volume in the left IPS have been reported in patients with comitant strabismus including IXT [44, 45]. Furthermore, patients with strabismic amblyopia exhibit impaired visuospatial function in the IPS [46]. Based on the previous evidence, we speculated that ocular misalignment interference may lead to impairment in the upstream dorsal pathway and eventually cause poor performance in visually-guided reaching and grasping [47].

Contrary to our findings, previous studies have shown that activation in the parietal cortex is enhanced during fusion tasks in patients with esotropia and exotropia [48, 49]. In addition, these studies did not find evidence of decreased activation in the patient group. Our 3D stimuli were composed of dynamic random dots that lacked any monocular depth cues. In contrast, the 3D stimuli in previous studies contained monocular cues or evoked vergence eye movement. Our results and the findings in previous studies [48, 49] imply that the effect of strabismus on stereoscopic processing is distinct from its effects on other neural circuits related to vergence control [50], depth cue integration [51], and attention [52].

Notably, a worse BF score was correlated with weaker cortical activation in dorsal visual and parietal areas in IXT patients. To our knowledge, this is the first study to identify correlations between cortical deficits and impaired stereopsis in patients with IXT. Dorsal visual areas including V3A, V7, and subregions of the IPS reportedly encode disparity magnitudes [9] and reliably distinguish among disparities over a wide range [8]. We inferred that hypoactivation along the occipital and parietal branches of the dorsal stream may be related to abnormalities in disparity magnitude discrimination. The cortical hypoactivation recorded in our study may reflect the severity of stereopsis impairment in patients with IXT.

Interestingly, earlier age at onset was correlated with reduced activation in the bilateral V3A in the patients before surgery. The development of stereopsis is constrained by a specific critical period from early infancy to nearly 5 years of age [53]. Accordingly, younger patients are more susceptible to anomalous binocular visual experience and suffer more severe deficits in stereopsis. We previously demonstrated that early age at onset is the only significant explanatory factor for reduced neural signals in the higher dorsal area of the parieto-occipital sulcus, based on multivariate regression analysis [17]. This regression finding suggested that earlier prolonged exposure to binocular misalignment might disrupt the normal development of stereopsis [17]. Taken together, our current results indicated that the development of stereoscopic processing is impaired by IXT.

In this study, patients before surgery showed hypoactivation of the bilateral LOC compared to the controls. The LOC is reported to be relevant to object-independent shape recognition. Single-cell evidence has shown that a number of LOC neurons exhibit a preference for disparity-defined convex or concave stimuli [54]. Preston and colleagues [9] reported that the representation of depth in the LOC makes categorical discrimination of crossed and uncrossed disparities. Therefore, higher-level ventral area LOC may use disparity-defined 3D information predominantly to facilitate object recognition [55]. Our finding suggests that stereoscopic processing in higher ventral areas experiences interference in IXT. Consistent with our results, the latest electrophysiological study demonstrated reduced excitatory binocular interactions in the LOC in patients with strabismus [41].

Furthermore, there was a significant association between the NCS and cortical response in the right LOC in patients with IXT. The NCS grades fusional control and reflects the severity of IXT [26]. Kourtzi and Kanwisher [56] reported functional MRI adaptation in the LOC when pairs of images presented successively had identical perceived shapes and different contours, but not when they had different shapes and identical contours. The similarity of shape detected in the LOC possibly guides strabismic suppression [56]. Using resting-state functional MRI, we previously reported that abnormal spontaneous neural activity in the right LOC is related to both binocular function and fusional control in patients with IXT [17]. Consistent with the prior evidence, dysfunction in the right LOC was associated with the deterioration of the disease in the present study.

Preoperative patients exhibited decreased activation in the left SEF, which is a cortical area within the dorsomedial frontal cortex that has been implicated in the higher aspects of oculomotor control [57] and visuomotor transformation [58]. The activity of neurons in the SEF is related to visually-guided saccades [59], smooth pursuit [60], performance monitoring [61], and eye-hand coordination [62]. Chan and colleagues [63] reported that gray matter volume in the SEF is increased in patients with comitant strabismus including IXT. In that study, the researchers regarded neuroanatomical abnormalities in frontal regions as compensation for cortical deficits during visual processing. Our results support this notion, highlighting the dysfunction in the SEF during visual processing.

Surprisingly, patients did not display significant differences in cortical activation after surgery compared to the preoperative values. We then divided patients after surgery into two subgroups according to their postoperative BF score. Subgroup results showed that cortical changes after surgery differ across individuals. The post-improved group had greater postoperative activation than preoperative activation in the right V3A and left IPS, while the post-stable group showed no difference between pre- and postoperative activation. The increased cortical activation in patients with improved stereopsis may reflect functional plasticity in the visual cortex [12]. It has been reported that interocular suppression and fixation instability may remain after surgical realignment and thus prevent the restoration of sensory function [64]. A prior psychophysical study demonstrated that sensory eye dominance is not improved by strabismus surgery, indicating that surgical realignment alone may not be sufficient for cortical adaptation [32]. Interestingly, there is evidence that comitant exotropia patients with improved stereopsis after surgery show significant functional and structural changes [12, 25], supporting the hypothesis that brain plasticity may contribute to the recovery of stereopsis. Based on previous findings and our subgroup results, we suggest that functional alterations after strabismus surgery differ across individuals and surgery alone may not be sufficient to induce functional plasticity in some patients. Previous prospective studies of perceptual training after surgery demonstrated more improvement in stereoacuity when compared with surgery alone [65, 66]. Therefore, the combination of surgical correction with some newly-developed strategies may be promising to promote functional plasticity in higher-level visual areas [24].

Correlation analysis showed that better postoperative stereoacuity was correlated with stronger cortical activation in the right V3A in the post-improved group, suggesting that functional plasticity of the visual cortex after strabismus surgery may underlie stereopsis improvement [67]. Human functional MRI studies have demonstrated that V3A has a higher sensitivity to disparity magnitude than other visual areas, containing organized structures correlated with stereoscopic perceptual judgments [39, 68, 69]. Chen *et al*. [70] recently illustrated that V3A makes a causal contribution to perceiving both stereoacuity threshold (the smallest detectable disparity) and the upper disparity limit (largest detectable disparity), and that stereoacuity threshold is affected after transcranial magnetic stimulation over area V3A. The phenomena reported in Chen’s study highlight the unique role of V3A in differentiating fine disparity signals. Considering the correlation between the enhanced neural response in V3A and the degree of stereoacuity, we postulate that V3A may be the key node associated with stereopsis restoration. Future studies focused on elucidating the involvement of V3A in stereopsis improvement are necessary for the development of new clinical strategies.

There were some limitations in this study. First, although we identified functional changes after surgery in patients with IXT, the follow-up period was limited to 6 months. A longer follow-up period (i.e., one or two years after surgery) is needed to explore long-term cortical plasticity, as well as its relationships with the prognosis of stereopsis and the recurrence of strabismus. Second, we only analyzed patients with a common type of exotropia. Future studies should explore neural mechanisms that underlie stereopsis impairment in patients with different types of strabismus (e.g., esotropia and exotropia) to extend the findings to a broader patient population.

To our knowledge, this is the first study to show relationships between cortical deficits and impaired stereopsis in patients with IXT. We demonstrated that decreased cortical activation along both the dorsal and ventral visual streams in IXT patients was correlated with worse stereopsis and poor fusional control and found strabismus surgery alone may not necessarily induce functional plasticity in stereoscopic processing. Our study further suggested that functional plasticity may underlie the improvement of stereopsis after surgery. Thus, additional postoperative strategies may be necessary to promote functional plasticity and enhance the restoration of stereopsis.

## Supplementary Information

Below is the link to the electronic supplementary material.

Supplementary file1 (PDF 346 kb)
