# Supplementary material for: Cortical Deficits are Correlated with Impaired Stereopsis in Patients with Strabismus
Source: Neurosci Bull. 2022 Dec 8;39(7):1039–49. doi: 10.1007/s12264-022-00987-7 (PMC10313621; doi:10.1007/s12264-022-00987-7)
Supplement: Supplementary file 1 — Supplementary file1 (PDF 346 kb) [file 12264_2022_987_MOESM1_ESM.pdf]

Supplementary Information

Supplementary Figures

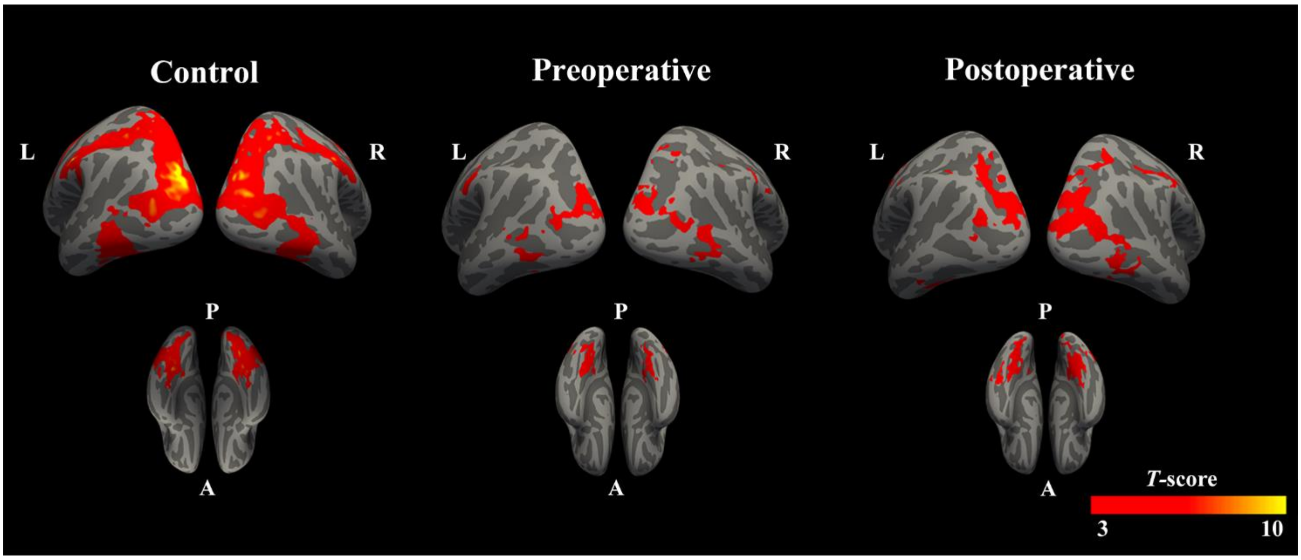

Fig. S1 Brain areas activated in response to disparity-varying 3D stimuli in the control and patient groups before and after surgery (preoperative and postoperative). Group mean activation maps for the control (left), preoperative (middle), and postoperative (right) groups are shown with lateral and ventral views of the left/right hemispheres. The lateral view is tilted by 45° for a better view. Color scale indicates *T*-scores (permutation test, FWE corrected $P < 0.05$). L, left; R, right; A, anterior; P, posterior.

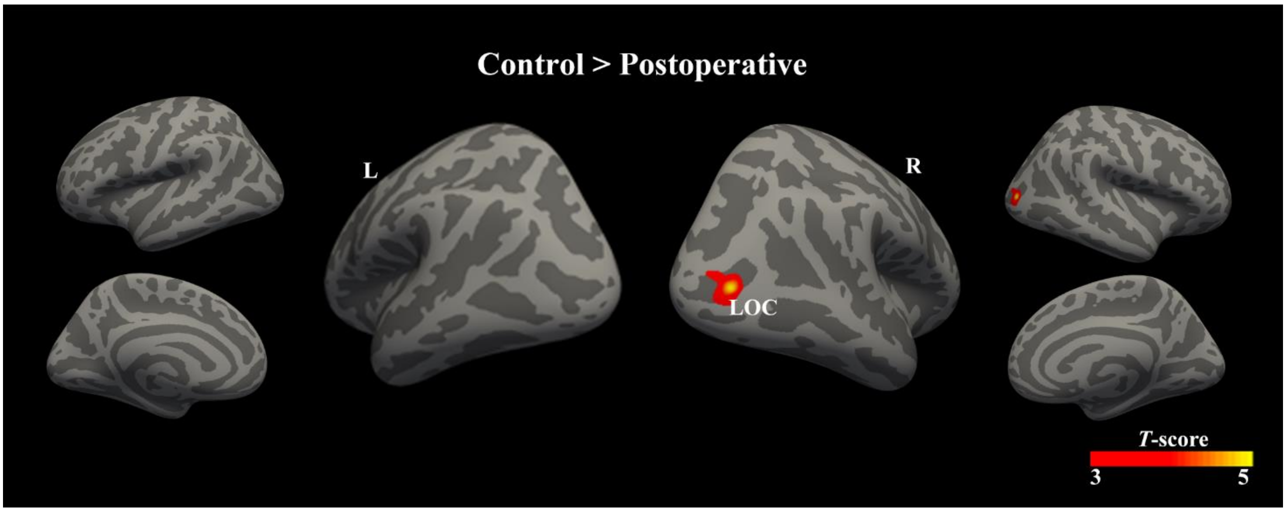

Fig. S2 Brain areas that exhibit significant differences in cortical activation between the patient group after surgery (postoperative) and the control group. When viewing disparity-varying 3D stimuli, postoperative patients display a decreased cortical response in the right LOC compared to controls. Color scale indicates *T*-scores (permutation test, FWE corrected $P < 0.05$). 3D, three-dimensional; LOC, lateral occipital cortex; R, right; L, left.

Supplementary Tables

Table S1 Modified binocular function score calculation

Near Stereoacuity	Binocular Function Score (near)
**Titmus Stereotest (arcsec)**	
40	1.6
50	1.7
60	1.8
80	1.9
100	2.0
140	2.1
200	2.3
400	2.6
800	2.9
**Stereo Fly Test**	
3552	3.6
**Worth 4-Dot Test**	
Fusion	4.0
Suppression	5.0

The binocular function score (1.6–3.6) was defined by the log value of the Titmus stereotest and stereo fly test.

Table S2 Demographic and clinical characteristics of patients before and after surgery

No.	Sex	Age	Onset	NCS	Deviation		BF Score		Subgroup[†]
					Pre	Post	Pre	Post	
1	M	27	24	4	35	2	2.3	2	Post-improved
2	F	21	10	3	40	5	3.6	1.6	Post-improved
3	F	13	12	5	30	0	2.3	1.6	Post-improved
4	M	25	7	5	60	8	2.6	1.6	Post-improved
5	F	22	12	8	66	10	2.1	1.9	Post-improved
6	M	26	17	3	77	8	2.6	1.8	Post-improved
7	F	18	8	6	70	6	2.3	2	Post-improved
8	F	28	15	6	53	4	2.6	1.7	Post-improved
9	M	27	17	6	40	6	4	1.7	Post-improved
10	F	18	12	7	55	6	2.6	1.6	Post-improved
11	F	29	13	9	45	10	5	5	Post-stable
12	M	26	20	3	30	5	2.3	2.3	Post-stable
13	M	29	13	3	72	8	2.9	2.9	Post-stable
14	M	14	11	5	26	0	2.1	2.1	Post-stable
15	F	14	4	7	25	10	5	5	Post-stable
16	M	30	9	6	40	16	2.6	2.3	Excluded
17	M	29	3	9	40	10	4	4	Excluded
18	F	26	5	6	56	18	2.6	2.6	Excluded

[†] Patients with successful surgical outcomes and improved BF score (#1–#10) were included in the post-improved group; five patients (#11–#15) with stable stereopsis (BF score) after surgery were included in the post-stable group. Three patients (#16–#18) with undercorrection after surgery were

excluded from the postoperative group, regardless of their postoperative stereopsis status. IXT, intermittent exotropia; Pre, preoperative; Post, postoperative; NCS, Newcastle Control Score; BF, binocular function.

Table S3 Differences in cortical activation between the patient group after surgery (postoperative) and the control group

	**Region**	**Hemi**	**Size (mm^2)**	**Peak MNI coordinate**			***T*-value**	**Cluster-wise**
				x	***y***	***z***		***P*-Value**
Control > Postoperative								
Ventral	LOC	R	297	35.7	−78.8	4.1	5.90	0.004

The statistical threshold was set as $P < 0.001$ at the voxel-level with FWE corrected $P < 0.05$ at the cluster-level, permutation test. Hemi, hemisphere; LOC, lateral occipital cortex; R, right; L, left; MNI, Montreal Neurological Institute.
